# A Scientometric Review on Mapping Research Knowledge for 3D Printing Concrete

**DOI:** 10.3390/ma15144796

**Published:** 2022-07-08

**Authors:** Chuan He, Shiyu Zhang, Youwang Liang, Waqas Ahmad, Fadi Althoey, Saleh H. Alyami, Muhammad Faisal Javed, Ahmed Farouk Deifalla

**Affiliations:** 1 School of Architecture, Changsha University of Science and Technology, Changsha 410015, China; zsy627377809@sina.com (S.Z.); liangyouwang66@sina.com (Y.L.); 2 Department of Civil Engineering, COMSATS University Islamabad, Abbottabad 22060, Pakistan; 3 Department of Civil Engineering, Najran University, Najran, Saudi Arabia; fmalthoey@nu.edu.sa (F.A.); shalsalem@nu.edu.sa (S.H.A.); 4 Structural Engineering and Construction Management Department, Faculty of Engineering and Technology, Future University in Egypt, Cairo 11835, Egypt; ahmed.deifalla@fue.edu.eg

**Keywords:** 3D printing, concrete, scientometric analysis, cementitious composites

## Abstract

The scientometric analysis is statistical scrutiny of books, papers, and other publications to assess the “output” of individuals/research teams, organizations, and nations, to identify national and worldwide networks, and to map the creation of new (multi-disciplinary) scientific and technological fields that would be beneficial for the new researchers in the particular field. A scientometric review of 3D printing concrete is carried out in this study to explore the different literature aspects. There are limitations in conventional and typical review studies regarding the capacity of such studies to link various elements of the literature accurately and comprehensively. Some major problematic phases in advanced level research are: co-occurrence, science mapping, and co-citation. The sources with maximum articles, the highly creative researchers/authors known for citations and publications, keywords co-occurrences, and actively involved domains in 3D printing concrete research are explored during the analysis. VOS viewer application analyses bibliometric datasets with 953 research publications were extracted from the Scopus database. The current study would benefit academics for joint venture development and sharing new strategies and ideas due to the graphical and statistical depiction of contributing regions/countries and researchers.

## 1. Introduction

Charles Hull, in 1986, initially introduced the 3D printing or additive manufacturing (AM) technology in stereolithography (SLA). Afterwards, it gained the attention of everyone, either from industry or an individual hobbyist [1]. The enhanced popularity of 3D printing is primarily because of its potentially freeform design, minimizing waste materials, mass customization, complex geometries manufacturing, and accelerating the fabrication procedure [2]. In the current era, the application of 3D printing technology in construction is becoming very prevalent [3,4]. Kim et al. [5] used the 3D printing technology to determine reinforced concrete beams’ shear strength having multiple interfaces before initial setting. Three-dimensional printing technology can offer new prospects in the construction sector, such as geometrical flexibility, labor cost reduction, safety and efficiency improvement, and hard/harsh area/environment construction [6,7]. The primary distinguishing component of 3D printing technology is the flexibility in geometry that enables the improved architectural appearance. Three-dimensional printing technology also offers the independency of shape on cost, ultimately providing design freedom [3]. Further, the additive/3D printing technology enables the creation of multi-functional components of a building and links the digital building and designing process [4,8]. The cost reduction, coupled with human resources, is also an essential component of said technology. It is linked with enhancement in safety and efficiency. Three-dimensional technology offers higher cost-effectiveness and accuracy with respect to traditional technology [4,9]. The need for formwork, a significant component in conventional construction, is also eliminated using 3D printing [4,10]. The enhancement in safety levels by reducing the injury rates can also be achieved by eliminating the formwork stage [10,11]. Furthermore, it also helps to reduce the on-site construction time [10]. The last and most important advantage of 3D printing is sustainability. The construction waste, specifically generated from formwork, is also significantly reduced by using this technology [3,10]. Initially, the lesser material would be consumed for casting and molding, followed by the possible optimization of construction provided by this technology and the reduction in materials consumed by this process itself. A further benefit of this technology also includes the reduction in transportation costs. In addition to that, this technology also comes up with reduced CO_2_ emissions by declining the inadequacies throughout the process of building. Multiple studies are going on for achieving sustainable development by using recycled/waste materials such as natural fibers, supplementary cementitious materials, construction and demolition waste, marble and ceramic waste powders, functionally graded materials etc., to conserve the natural resources [12,13,14,15,16,17,18,19,20,21,22]. 

Hence, it can be concluded that the rising agreement of using the 3D printing technology over conventional methods is due to multiple benefits such as highly accurate complex geometry fabrication, design flexibility, personal customization, and maximum material conservation. A wide variety of materials are applied in 3D printing such as, concrete, polymers, metals, and ceramics. Acrylonitrile butadiene styrene (ABS) and polylactic acid (PLA) are among the significant polymers that are utilized for composites 3D printing. Advanced alloys and metals are usually used in aerospace to reduce the time and cost consumption involved in conventional methods. Three-dimensional printing of scaffolds mainly consumes ceramics, whereas concrete is the primary material for building additive manufacturing. However, large-scale printing is still quite limited due to poorer anisotropic behavior and mechanical characteristics of the 3D printed parts. Accordingly, there is a need to have an optimized 3D priming pattern to restrict anisotropic behavior and error sensitivity [23]. 

The finished products quality is also dependent on the printing environment [24]. The multiple sizes, i.e., micro to macro scale, of parts fabrication can be performed by using additive manufacturing (AM). Whereas the printed parts accuracy is mainly dependent on the precision of the applied printing scale and method. For example, 3D printing at the micro level offers challenges with the layer bonding, surface finish and resolution that usually need sintering like post-processing treatments [25]. The limited 3D printing materials provide challenges in employing 3D printing technology in different industrial sectors. Therefore, appropriate materials are needed to be utilized in 3D printing. In addition, the improvement techniques for 3D printed parts’ mechanical characteristics are also to be developed [26].

The enhancement in additive manufacturing leads to the development of research on 3D printing concrete. The obstacles in scholarly collaboration and creative investigation are created due to researchers’ information restraints. Accordingly, the creation and application of a process for the researchers/scientists to obtain important information from dependable sources are vital. Applying a scientometric method via the software may support overcoming this loophole and research gap. The main aim of the current study is to provide a detailed review of 3D printing methods with a focus on utilized materials, primary techniques applied, their applications, and the current state in different industry sectors. The research challenges and gaps in accepting 3D technology are also provided in this paper. The scientometric analysis of research published in 3D printing concrete up to 2022 is intended in this study. The quantitative evaluation of the bulk research dataset may be undertaken with the help of scientometric analysis using appropriate software [27,28]. The traditional review natured studies are somehow weak in their respective capability of connecting the various literature segments thoroughly and accurately. Co-occurrence, science mapping, and co-citation are key factors of exploration in the current era [29,30,31]. Identifying sources with co-occurrence of keywords, most research articles, the main credible researchers in terms of citations and papers, and actively engaged research areas in 3D printing concrete can also be performed with scientometric analysis. The bibliometric data of 953 related research articles is extracted using the Scopus dataset, which is determined afterwards using VOSviewer. The current study would assist academics in the engineering field belonging to various geographical locations in exchanging ground-breaking novel methods/ideas, creating joint ventures, and forming research alliances due to the graphical and statistical depiction of countries and authors.

## 2. Methodology

In this study, the scientometric analysis is carried out for the research dataset to evaluate the different aspects of bibliographic data [32,33,34,35,36]. Multiple studies have been conducted and reported on this matter depicting the questionable application of a reputable search engine. The two highly precise search engines, i.e., Web of Science and Scopus, are specifically explored for said aim [37,38]. The research data to conduct the current study on 3D printing concrete were collected using the academically highly recommended search engine, i.e., Scopus [39,40]. As of May 2022, the Scopus search for “3D printing concrete” found 1837 articles from 1998 to 2022. Multiple filters, depending upon preferences, are applied to avoid unnecessary data. The “journal research article”, “journal review”, “conference review”, and “conference paper” are opted as the document type. The “source type” selected is “conference proceeding” and “Journal”. The chosen period restriction for “publication year” is set to “2022”, and “English” is set as “language” constraint. For more scrutiny, the “engineering”, “environmental science”, and “material science”, are selected as “subject areas”. Following the employment of said desirables, a total of 953 records are kept. Similarly, multiple studies have been conducted by using same method [41,42,43,44,45].

**Figure 1:**
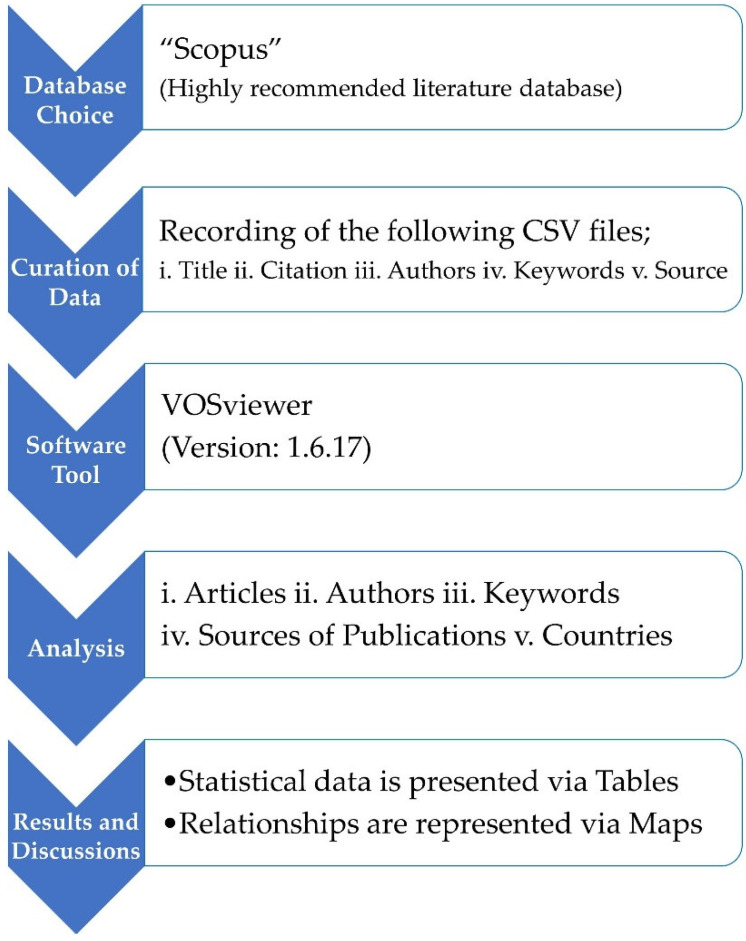
Sequential research methodology.

In academics, scientific mapping is developed to analyze bibliometric data, which is usually employed to analyze scientometric inquiries [46,47,48]. Comma separated values (CSV) files are used to save Scopus records for further determination with the help of a suitable software tool. The quantitative evaluation of the recovered records’ literature and scientific visualization are generated using VOSviewer (version: 1.6.17). In academics, the VOSviewer is a majorly suggested and mainly used tool over a broader range of areas, and this mapping tool (open-source) has easy availability [49,50,51,52]. Therefore, the application of VOSviewer in the current study satisfies its goals. Loading of attained CSV files in VOSviewer is performed, and further evaluation is conducted to retain the consistency and integrity of data. At the same time, assessment of bibliographic data, countries’ participation, the publication sources, the researchers having more citations and publications, the frequently appearing keywords, and the country’s involvement are assessed. The various aspects and their co-occurrence and relationships are graphically represented, whereas the figures’ statistics are listed in tables. Figure 1 presents the strategical flowchart for scientometric analysis.

## 3. Analysis of Results

### 3.1. Annual Publications and Related Subject Areas 

**Figure 2:**
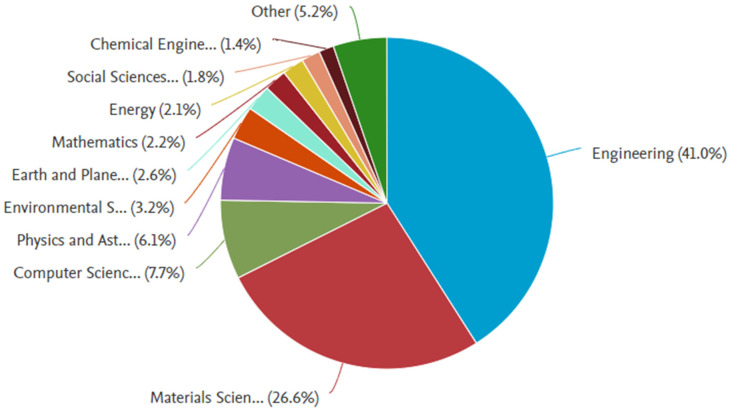
Articles subject area.

**Figure 3:**
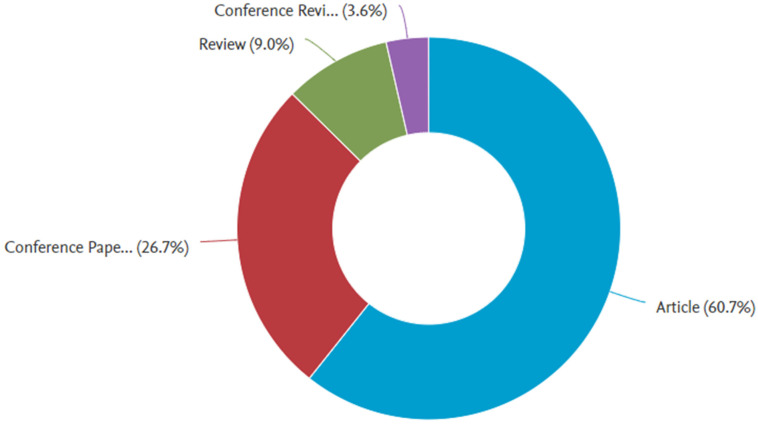
Published document types in the relevant field of study.

**Figure 4:**
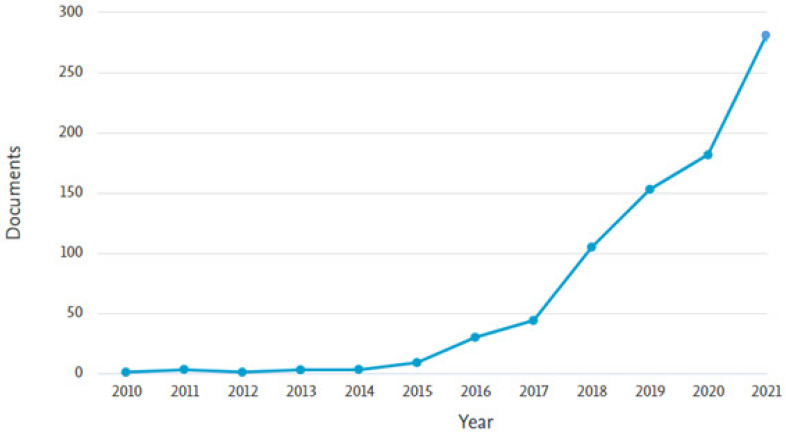
Annual publication trend of articles.

The analysis for discovering the most appropriate research areas is carried out by applying the Scopus analyzer. The three leading articles producing sections are engineering, materials science, and computer science, having almost 41%, 27%, and 8% articles, respectively, bearing the overall contribution of 76% depending on document count, as presented in Figure 2. Furthermore, Figure 3 shows the evaluation of paper type in the Scopus database for searched terms. According to the current study, conference review papers, journal review articles, and journal articles bear around 3%, 9%, 27%, and 61% of documents. The annual publication trend in the current research field from 1998 to 2022 is shown in Figure 4, as the occurrence of the first respective article was revealed in 1998. A mild rise in publication trend for the said research area, i.e., 3D printing concrete, is observed, with an approximate average of three annual articles until 2014. Afterwards, a gradual rise in annual publications is observed, with an approximate average of twenty articles per annum from 2015 to 2019. However, a significant enhancement in annual publications has been observed during the last three years, (i.e., 2020–2021). Recently, a drastic increase in 3D printing concrete for building and concrete research has been observed, depicting the initiative for all-rounded and comprehensive research work in the said field [53]. Scientific research globalization might be the reason behind increasing trend development in 3D printing concrete.

### 3.2. Publication Sources

**Figure 5:**
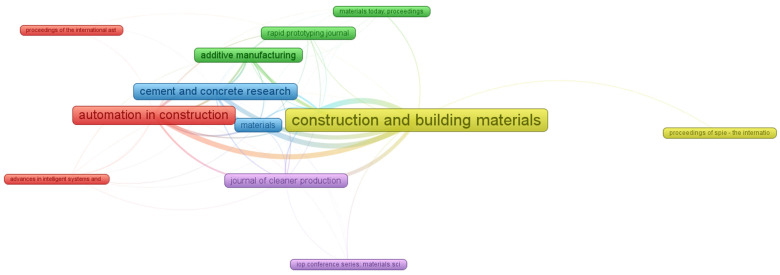
Scientific visualization of publication sources with at least 10 publications in the related research area.

The VOSviewer was utilized on the gathered bibliographic database to evaluate the published sources. While performing analysis, the sources are taken as “unit of analysis”, whereas the “bibliographic coupling” is opted as a “kind of analysis”. The minimum quantum of articles per source is set to ten. The sources of publication that met the said requirement are 14 out of 265. The publication sources are listed in Table 1, with at least ten published articles presenting data on 3D printing concrete until 2022 and the citation’s quantum acquired in the said period. The three main journals/sources, depending upon the paper count, are i. “Construction and Building Materials” having 60 papers, ii. “Additive Manufacturing” has 39 documents, and iii. “Automation in Construction” with 35 articles. Furthermore, the primary three sources having overall maximum citations are “Automation in Construction”, “Additive Manufacturing”, and “Buildings”, born 1580, 871, and 798 citations, respectively. Automation in construction also covers the aspect of multiple software applications such as building information modelling (BIM) for 3D printing concrete [54]. The coupling of 3D printing concrete with BIM to monitor and track novel variables was also performed by Azhar [55] and Bryde, et al. [56]. Combining 3D printing and BIM may make the creation of customized building components easier and facilitate sophisticated and complex design [53]. Davtalab, Kazemian and Khoshnevis [54] also declared that robotic construction is a construction industry revolution by using 3D printing concrete. This significant research exploration in the area of 3D printing concrete is come out to be the reason for intended scientometric analysis in the said research area. Further, conventional review studies are not enough to develop scientific visualization maps. The mapping journals with a minimum of ten articles in understudied research areas is presented in Figure 5. The quantum of research in 3D printing concrete in the form of articles is directly proportional to the size of the box showing the impact of the journal. The bigger the dimension of the box, the effect is more superior. For example, the biggest box in terms of sizes is for “Construction and Building Materials” showing the significant importance of this source in the considered field. Based on the type, five groups are developed, and all of them are offered in different colors, i.e., purple, red, green, blue, and yellow. The formation of groups is based on the similar article co-citations frequency [57]. The patterns of published articles’ co-citation are the basis of group creation in VOSviewer. For example, the red group comprises three sources having frequent co-citations in similar works. In addition, the space among frames/journals in a group shows significant relationships compared to the other far-spaced frames. For example, “Additive Manufacturing” is more firmly correlated with “Rapid Prototyping Journal” than with “Materials Today: Proceedings”.

### 3.3. Keywords

**Figure 6:**
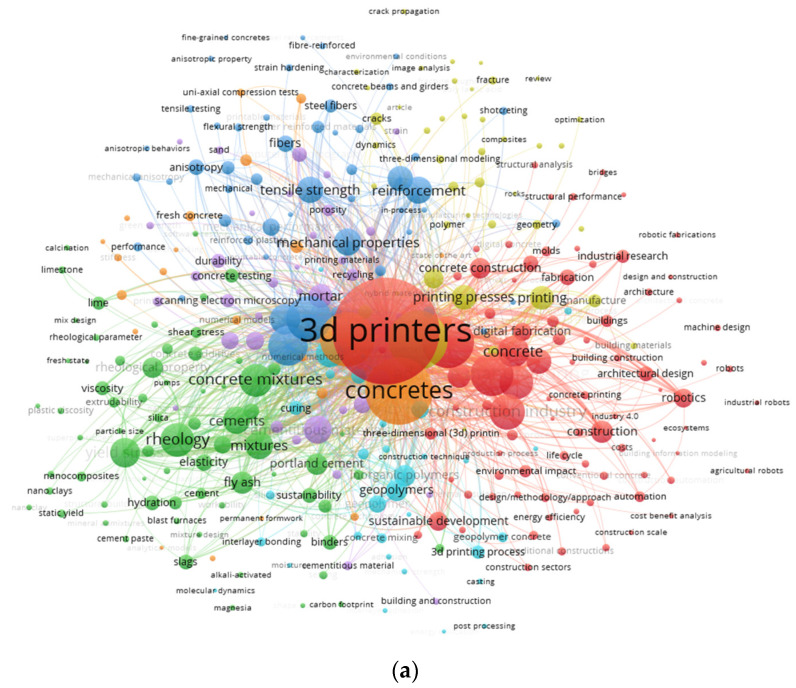

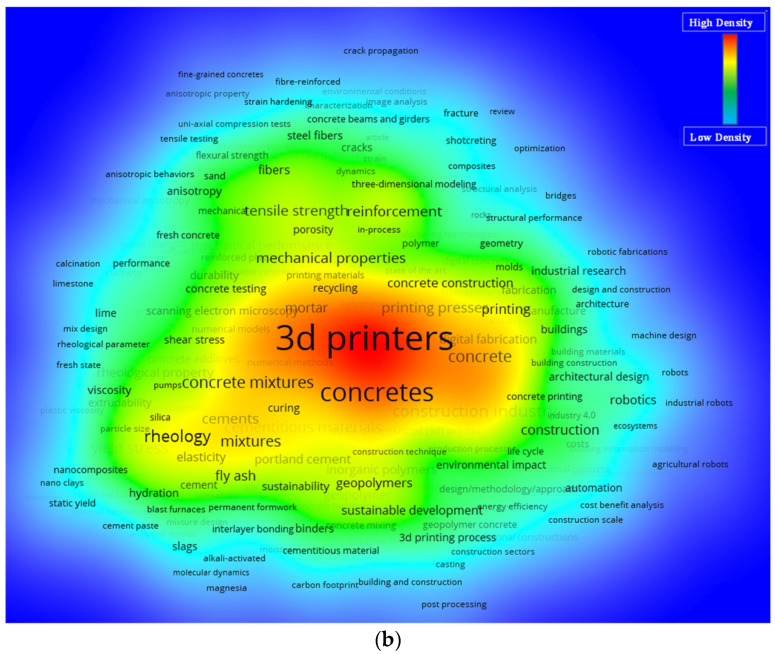
Analysis of keywords; (**a**) scientific visualization and (**b**) density visualization.

The fundamental subject of a study domain is highlighted and defined with the help of keywords in the research [58]. For the evaluation, the “analysis type” is selected as “co-occurrence”, whereas the “analysis unit” is opted to “all keywords”. The minimum repetition restriction is set at 20 for a keyword. Accordingly, 96 keywords out of 4185 are taken. The leading and most frequently used 20 keywords in published papers on relevant research areas are provided in Table 2. The terms 3D printers, concretes, 3-D printing, 3D printing, and concrete printings are among the most frequent five keywords in the considered area of research. As per the analysis of keywords, 3D printing concrete has been mainly studied for concrete mixtures, its rheology, and mechanical properties. Furthermore, it has also been explored for multiple types of building systems. Duballet et al. [59] classified the 3D printing concrete building systems based on five parameters: extrusion scale, object scale, printing environment, assembly parameter, and printing support. This classification was mainly featured for reinstating techniques apart from a single extrusion phase for concrete 3D printing at a larger scale. The keywords visualization map in terms of linkages, co-occurrences, and the occurrence frequency-related density is shown in Figure 6. The frequency of keywords is depicted by the size of the circle for the respective keyword, while the co-occurrence in papers is shown by its position (Figure 6a). It is evident from the graph that the comparatively bigger circles are for leading keywords depicting their significance for research on 3D printing. The formation of groups is also made for keywords to reflect the keywords’ co-occurrence over several research publications. The multiple keywords’ co-occurrence in published articles is the basis of color-coded grouping. Four different colors, i.e., green, red, yellow and blue, indicate the group’s existence (Figure 6a). The concentrations for density of keywords are indicated by different colors (Figure 6b). The colors are aligned with respect to respective density concentrations. The red color shows the highest, whereas the blue color shows the lowest density concentration. Three-dimensional printers and concretes show red symbols depicting significant density concentration. This finding may aid ambitious researchers in selecting keywords that would enable the published data identification in a specific area.

### 3.4. Authors

**Figure 7:**
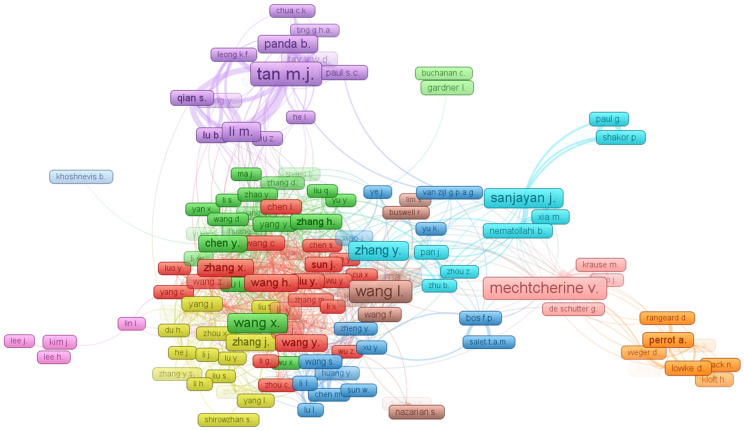
Scientific visualization of researchers who published articles in relevant research area.

A researcher’s influence in a specific study area is depicted from the citations [60]. Accordingly, the “co-authorship” is selected as a “kind of analysis”, whereas; “authors” is chosen as the “unit of analysis” for the authors’ assessment. The efficacy of a researcher is hard to determine while considering all parameters, such as total citations, the number of publications, and average citations. Contrary to this, a researcher’s evaluation is performed by considering each factor independently, i.e., total citations, total publications, and average citations. The leading researcher is Tan, M.J., having 34 publications, followed by 29 publications each by Panda, B. and Mechtcherine, V. Afterward, Sanjayan, J. and Ma, G. are prominent, with 28 publications each. However, in terms of total citations, Tan, M.J leads the field with 2453 citations, followed by Panda, B. having 2362 citations in the 3D printing concrete research area. In addition, upon comparing the citations average, Paul, S.C. stands out with an average of 113, followed by Panda, B. having an average of 81 and Tan, M.J with a 72 average. The correlation between most eminent researchers and authors with a minimum of 10 publications is illustrated in Figure 7. The noticed largest network of interconnected researchers is seven. It is revealed from this analysis that a few researchers are inter-connected in terms of citations in the 3D printing concrete research area.

### 3.5. Articles

**Figure 8:**
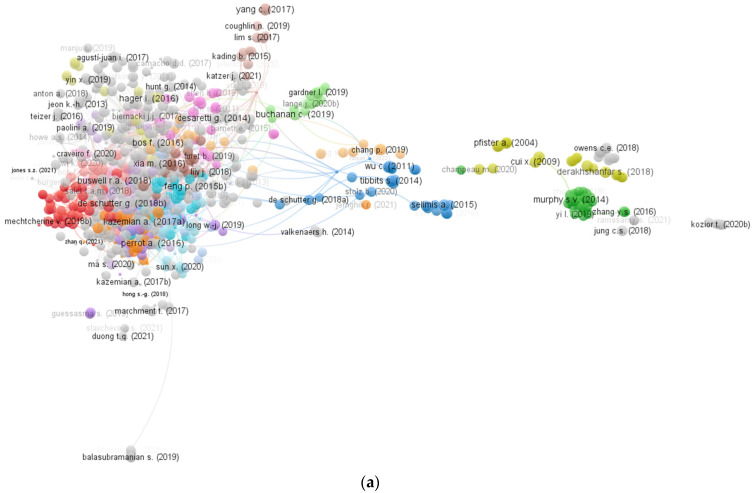

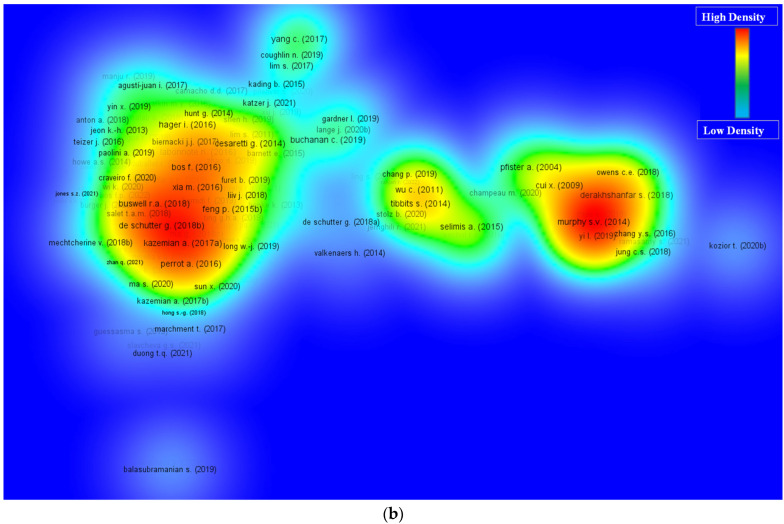
Published articles scientific mapping in relevant area of research until 2022; (**a**) connected documents based on citations (**b**) connected articles density.

The number of article citations influences a specific research area. Articles with a higher citation count are known as pioneers in relevant research areas. To evaluate articles, the “bibliographic coupling” is set for “kind of analysis”, and “documents” is designated as “unit of analysis”. The set limitation of most minor citations for an article is 50. In the 3D printing concrete research area, the top 10 articles, as per citations, are presented in Table 3 with respective citations and authors’ detailing. Ngo, Kashani, Imbalzano, Nguyen and Hui [26] have 2520 citations for the research article titled; “Additive manufacturing (3D printing): A review of materials, methods, applications and challenges”. For their relevant publications, Stansbury and Idacavage [61] and Buswell et al. [62] have 793 and 466 citations, respectively, and are placed in the first three positions. Furthermore, the linked articles mapping, based on citations and their density in the considered area, is shown in Figure 8. The inter-connected articles citation mapping is presented in Figure 8a, whereas, in Figure 8b, the enhancement of density concentration by top articles is revealed from the density mapping.

### 3.6. Countries

**Figure 9:**
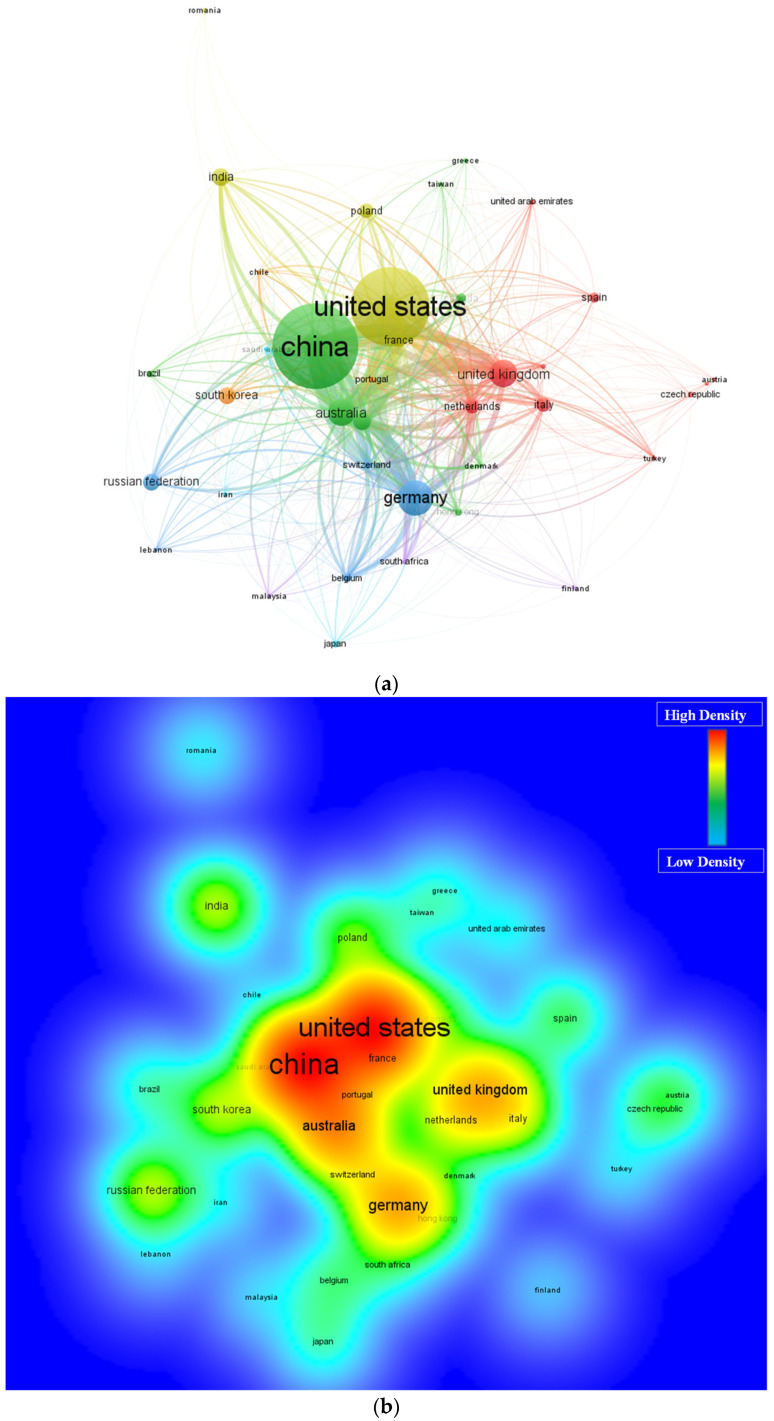
Scientific visualization regions having minimum ten publications in relevant research area until 2022 (**a**) network visualization and (**b**) density visualization.

The contribution of multiple countries is comparatively more towards 3D printing concrete research than others, and different expectations are there for enhancement in contribution. A network map is developed to help researchers access the areas related to 3D printing concrete research. Again, “Bibliographic coupling” is taken as a “kind of analysis”, whereas, “countries” are opted for as a “unit of analysis”. The limitation of the minor article for a nation is set at 10, and the countries met the desired limitation are 38 (Table 4). China, the United States, and Germany have the most articles with 377, 348, and 148 documents. Furthermore, the top three countries with the most considered research area citations of 10,514, 6179, and 3435 are the United States, China and Australia. The science mapping visualization and nation density inter-connected with citations is illustrated in Figure 9. The box size is directly proportional to a country’s effect on the considered area of research (Figure 9a). The most engaging countries have more density, as illustrated in the map of density visualization (Figure 9b). It may be noted that the publication trend in developed countries such as the USA, China, Australia, Germany and UK is significantly more than that in developing countries such as India, Pakistan, etc. [69]. As in developed countries, there are diverse applications of 3D printing; however, in recent years, this technology is also gaining attention in countries. There is a huge potential for 3D printing in developing countries [70,71]. The graphical and statistical analysis of the contributing countries may help concerned scientists form joint ventures, develop scientific alliances, and exchange novel ideas and methods. Scientists from different countries contributing for enhancing research on 3D printing concrete may collaborate with specialists in the said research area and yield from their expertise.

## 4. Discussions and Future Perspectives

The mapping and statistical overview of different aspects of the 3D printing concrete literature are presented in the current study. The conventional and manually conducted review studies have limited capability in terms of comprehensiveness and precise inter-connectivity among the various literature segments. The identification of most articles publishing journals, the frequently applied/used keywords in articles, countries having significant contributions, and authors and articles with most citations in the research field of 3D printing concrete is made in the current study. It is revealed from the keyword analysis that 3D printing concrete has been mainly explored in terms of its mechanical and rheological properties [72,73,74,75,76]. Furthermore, 3D printing is also researched for manufacturing geopolymer concrete [76,77,78]. Three-dimensional printing has various benefits upon utilization as concrete. The new prospects that can be utilized by 3D printing construction such as labor cost reduction, geometrical flexibility, efficiency improvement, safety and hard area construction [6,7]. In addition, physical construction consumes a bulk quantity of energy that comes out with higher CO_2_ emissions [79]. As a result, there are rising concerns regarding natural resource depletion. Thus, 3D printing concrete reduces the cement requirement, resulting in sustainable construction with reduced CO_2_ emissions [80,81,82]. The application of 3D printing concrete may also have resolved difficulties in waste management, specifically in the formwork [3,10]. The above-mentioned 3D printing concrete applications are yet in the phase of development. Detailed analyses are still required before their application broadens. Presently, the available research on 3D printing concrete is mainly based on their insight for extracting the optimal dosage of mix ingredients for desirable properties. Additionally, due to inferior properties and anisotropic behavior, the applicability of 3D printing at a larger scale is restricted. Therefore, it can be said that the information in said field is developing yet and needs to pass specific transition stages to accomplish optimum commercial applications and replace conventional manufacturing techniques. Therefore, the following research horizons in the field of 3D printing concrete may further be explored:

- Three-dimensional printed components’ structural integrity, especially in regions vulnerable to natural disasters, seismic activity, military attacks and extreme climatic conditions, needs to be ensured by performing structural testing and developing specified standards and codes.
- Due to the provision of controlled environmental conditions for performing the experiment on 3D printed components, its behavior may not depict the true performance. As in real site conditions, the components are exposed to variable climatic factors such as humidity, temperature and precipitation, debris and dust, and varied lighting, etc. [83]. Therefore, the performance of 3D printing concrete components may be evaluated under real environmental conditions to ensure the global efficiency of this method.
- The effective implementation of the 3D printing approach is dependent on reliable, strong and printer-compatible materials [84,85]. Hence, the in situ materials that are available locally should be used for printing to have compatible and effective printing.
- Further research should also be conducted for large-scale building construction and experimentation to ensure the real capacity of 3D printing technology and to depict the application of this technology in the industry.
- Furthermore, nowadays, calcium carbonate (CaCO_3_) whisker is used as micro-fiber in cementitious composites to improve the micromechanical properties of concrete [86,87,88,89,90,91,92,93,94]. Hence, the exploration of CaCO_3_ whisker for 3D printing concrete would be an interesting horizon to explore.
- Three-dimensional printing is still a new technology; therefore, the information on its life cycle cost, including the maintenance and upfront costs, is limited yet. Further, there may be variations in the costs of design, planning, machinery, labor, and materials from country to country [95]. Therefore, a thorough life cycle cost analysis should be conducted for 3D printing technology to have detailed insight into its cost–benefit ratio with respect to conventional construction [96].
- Furthermore, the information regarding the life cycle assessment (LCA) of 3D printing concrete is also limited and demands thorough exploration in terms of its sustainability impact, preparation of material, construction, utilization, and ultimately the structures’ demolition. This information is necessary to explore to have a clear picture of 3D printing concrete environmental impacts [97,98].

## 5. Conclusions

The abundance of scientific information produced in recent years, along with new communication channels, prompted the research community to propose the metric that gave origin to the new field of bibliometrics. This utilizes mathematical and statistical analysis techniques that permit getting dependable quality indicators. Thus, it is feasible to determine the number of documents published by an institution, nation, research group, or individual with the highest scientific output. A bibliometric study is an appropriate tool for identifying the volume and growth trend of literature focusing on concrete for the further 3D printing-related investigation that would be helpful for early-stage researchers.

The main aim of the current study is to perform a scientometric analysis of the literature available on 3D printing concrete to assess different measures. The Scopus database is enquired for 953 related articles, and the outcomes are evaluated by applying the VOSviewer program. It is revealed from the conducted analysis that the top three journals are “Construction and Building Materials”, “Additive Manufacturing”, and “Automation in Construction”, having 60, 39, and 35 articles, respectively. Further, the top three journals having the most citations of 1580, 871, and 798 are “Automation in Construction”, “Additive Manufacturing”, and “Buildings”, respectively. The analysis of keywords regarding the considered research area depicts that 3D printers, concretes, 3D printing, 3-D printing and concrete printing are the five most frequently appearing keywords. The keyword analysis revealed that 3D printing is mainly explored as concrete in the construction industry.

The top researchers are also classified based on the number of citations, publications, and average citations. Tan, M.J, with 34, Panda B., and Mechtcherine, V., with 29 each, and Sanjayan, J., and Ma, G., with 28 articles each, are among the leading three researchers with the most publications. With 2453 citations, Tan, M.J. leads the field, followed by 2362 citations of Panda, B. and 1441 citations o Bos, F.P. untill 2022. Furthermore, comparing average citations, the stand-out authors are Paul, S.C., who has almost 113, Tay, Y.W.D., who has around 95, and Panda, B., who has 85 average citations. In the analysis of articles related to 3D printing concrete, Ngo, Kashani, Imbalzano, Nguyen and Hui [26] have 2520 citations for the article “Additive manufacturing (3D printing): A review of materials, methods, applications and challenges”. Stansbury and Idacavage [61] and Buswell, De Silva, Jones and Dirrenberger [62] have 793 and 466 citations for the respective publications and are among the best three.

The leading countries are also determined by their contribution to the 3D printing concrete research area. China, the United States, and Germany have contributed 377, 348, and 148 articles. Further, the countries, i.e., the United States, China, and Australia, have received citations of 10,514, 6179, and 3435, respectively. The 3D printing concrete application in the construction industry would develop sustainable construction by having reduced demand for cement, waste, and formwork requirements, ultimately saving natural sources and declining CO_2_ emissions. The applicability of 3D printing concrete at a larger scale is still quite limited, and most of its applications are under exploration. Further analysis is also vital for broadening the effective applications of 3D printing concrete.

## Figures and Tables

**Table 1:** Sources of publication having minimum 10 publications in the considered research area till 2022.

S/N	Publication Source	Number of Publications	Total Number of Citations
1	*Automation in Construction*	35	1580
2	*Additive Manufacturing*	39	871
3	*Buildings*	13	798
4	*Lecture Notes in Civil Engineering*	22	727
5	*Materials and Design*	12	716
6	*Advanced Functional Materials*	12	381
7	*Construction and Building Materials*	60	309
8	*Cement and Concrete Research*	20	307
9	*Cement and Concrete Composites*	18	165
10	*Journal of Building Engineering*	12	131
11	*Rapid Prototyping Journal*	22	115
12	*Polymers*	10	74
13	*3D Printing and Additive Manufacturing*	11	71
14	*Materials*	45	56
15	*Applied Sciences (Switzerland)*	12	56

**Table 2:** Fifty leading frequently used keywords in 3D printing concrete research.

S/N	Keyword	Occurrences
1	3DPrinters	558
2	Concretes	272
3	3-D Printing	209
4	3D Printing	188
5	Concrete Printings	184
6	3D Concrete Printing	154
7	Additive Manufacturing	139
8	Compressive Strength	104
9	Construction Industry	89
10	Additives	85
11	Rheology	81
12	Concrete	75
13	3D-Printing	70
14	Concrete Mixtures	70
15	Extrusion	66
16	Yield Stress	66
17	Reinforcement	61
18	Cementitious Materials	60
19	Cements	58
20	Reinforced Concrete	57
21	Mechanical Properties	55
22	Printing	51
23	Mixtures	50
24	Mortar	50
25	Tensile Strength	50
26	Concrete Products	48
27	Concrete Industry	47
28	Construction	45
29	Concrete Construction	39
30	Fly Ash	37
31	Rheological Property	37
32	Portland Cement	36
33	Geopolymers	35
34	Inorganic Polymers	35
35	Digital Fabrication	34
36	Structural Design	34
37	Buildability	32
38	Digital Construction	30
39	Sustainable Development	30
40	Mechanical Performance	28
41	Geopolymer	27
42	Hardening	27
43	Shrinkage	26
44	Fabrication	25
45	Cement Based Material	24
46	Concrete Buildings	24
47	Fibers	24
48	Anisotropy	23
49	Binders	23
50	Concrete Additives	22

**Table 3:** Top 10 highly cited published articles up to 2021 in the research of RHA concrete.

S/N	Article	Title	Total Number of Citations Received
1	Ngo, Kashani, Imbalzano, Nguyen and Hui [26]	Additive manufacturing (3D printing): A review of materials, methods, applications and challenges	2520
2	Stansbury and Idacavage [61]	3D printing with polymers: Challenges among expanding options and opportunities	793
3	Buswell, De Silva, Jones and Dirrenberger [62]	3D printing using concrete extrusion: A roadmap for research	466
4	Bos, et al. [63]	Additive manufacturing of concrete in construction: potentials and challenges of 3D concrete printing	453
5	Gosselin, et al. [64]	Large-scale 3D printing of ultra-high performance concrete—a new processing route for architects and builders	424
6	Perrot, et al. [65]	Structural built-up of cement-based materials used for 3D-printing extrusion techniques	384
7	Tay, Panda, Paul, Noor Mohamed, Tan and Leong [53]	3D printing trends in building and construction industry: a review	310
8	De Schutter, et al. [66]	Vision of 3D printing with concrete—Technical, economic and environmental potentials	305
9	Kazemian, et al. [67]	Cementitious materials for construction-scale 3D printing: Laboratory testing of fresh printing mixture	285
10	Wolfs, et al. [68]	Early age mechanical behaviour of 3D printed concrete: Numerical modelling and experimental testing	284

**Table 4:** Leading countries in published articles 3D printing concrete research area until 2022.

S/N	Country	Number of Publications	Total Number of Citations
1	China	377	6179
2	United States	348	10,514
3	Germany	148	2813
4	United Kingdom	114	2540
5	Australia	113	3435
6	Singapore	72	2725
7	India	70	433
8	Russian Federation	69	132
9	South Korea	67	1268
10	France	58	1807
11	Italy	57	1022
12	Netherlands	56	1958
13	Poland	56	466
14	Spain	41	362
15	Canada	37	978
16	Belgium	30	698
17	Brazil	28	338
18	Japan	28	502
19	Switzerland	28	1557
20	Hong Kong	26	220
21	Czech Republic	25	113
22	Portugal	23	251
23	South Africa	21	382
24	Norway	20	540
25	United Arab Emirates	20	279
26	Romania	18	48
27	Taiwan	17	111
28	Greece	16	400
29	Iran	16	228
30	Turkey	15	50
31	Austria	14	70
32	Malaysia	13	110
33	Denmark	12	518
34	Sweden	12	60
35	Finland	11	56
36	Chile	10	35
37	Lebanon	10	119
38	Saudi Arabia	10	229

## Data Availability

The data used in this research has been properly cited and reported in the main text.
